# An impact of lipid profile and lipid lowering drugs on ≥70 year olds of an upper socioeconomic class: a retrospective cohort study

**DOI:** 10.1186/s12944-021-01529-2

**Published:** 2021-09-29

**Authors:** Yehudit Eden Friedman, David M. Steinberg, Michal Canetti, Ido Cohen, Shlomo Segev, Ophira Salomon

**Affiliations:** 1 Internal Medicine Department E, Sheba Medical Center, Tel Hashomer, Israel; 2 Sackler Faculty of Medicine, Tel Aviv University, Tel Aviv, Israel; 3 Department of Statistics and Operations Research, Faculty of Exact Sciences, Tel Aviv University, Tel Aviv, Israel; 4 Institute of Medical Screening, Sheba Medical Center, Tel Hashomer, Israel; 5 Thrombosis Unit, Sheba Medical Center, Tel Hashomer, Israel

**Keywords:** Elderly, Low density lipoprotein cholesterol, Lipid lowering drugs

## Abstract

**Background:**

Life expectancy has greatly increased, generating an improvement in screening programs for disease prevention, lifesaving drugs and medical devices. The impact of lowering low-density lipoprotein cholesterol (LDL-C) in the very elderly is not well-established. Our aim was to explore the association of LDL-C, high density lipoprotein cholesterol (HDL-C) and lipid lowering drugs (LLDs) on cognitive decline, malignancies and overall survival.

**Methods:**

This was a retrospective cohort study. Our study comprised 1498 (72.7%) males and 561 (27.3%) females, aged ≥70 who had attended the Institute for Medical Screening (IMS), Sheba Medical Center, Israel at least twice during 2013–2019. Data were obtained from the computerized database of the IMS. A manual quality control to identify potential discrepancies was performed.

**Results:**

Overall, 6.3% of the subjects treated with LLDs (95/1421) versus 4.2% not treated (28/638), cognitively declined during the study years. No statistically significant effects of LDL-C, HDL-C and LLDs on cognitive decline were observed after correcting for age, prior stroke and other vascular risk factors. With regard to cancer, after adjusting for confounders and multiple inferences, no definite relationships were found.

**Conclusions:**

This analysis of an elderly, high socioeconomic status cohort suggests several relationships between the use of LLDs and health outcomes, some beneficial, especially, with regard to certain types of cancer, but with a higher risk of cognitive decline. Further studies are warranted to clarify the health effects of these medications in the elderly.

**Supplementary Information:**

The online version contains supplementary material available at 10.1186/s12944-021-01529-2.

## Background

Continual improvement in medical and living standards has increased amongst the elderly population who now live > 90 years of age. Abnormalities in cholesterol metabolism significantly affect human survival [1]. Increased low-density lipoprotein cholesterol (LDL-C), an established risk factor for cardiovascular disease (CVD), is often treated by lipid lowering drugs (LLDs) to prevent cardiovascular events. However, the effect of LLDs amongst patients > 70 years old is less evident, with only a few trials having investigated this population [2, 3]. Moreover, there is a subset of elderly people with high LDL-C who live longer than those with low levels [4]. Another potential beneficial effect of increased LDL-C is the inactivation of microorganisms which may explain the reduced mortality from respiratory and gastrointestinal diseases [5, 6]. High LDL-C may also play a protective role against cancer, as witnessed by lower cancer mortality in patients with familial hypercholesterolemia.

A strong inverse correlation was found between high-density lipoprotein cholesterol (HDL-C), all-cause and cardiovascular mortality [7]. HDL-C and total cholesterol have been reported as inversely correlated with lung, breast, colon cancer [8–11] and cancer survival [12]. Notably, high triglycerides (TGs) were found to positively correlate with the incidence of lung, esophageal and colon cancer [13]. The role of statins in the elderly is still under debate [14–17]. New onset of diabetes is also of concern [18]. In this study, we investigated the potential association of lipid profile, treatment with LLDs and morbidity of older individuals with a high socio-economic status (SES).

## Methods

### Study population

Our study population comprised patients born in 1949 or earlier, who had reached aged ≥70 by the end of 2019 when the study was terminated, and who had attended the Institute for Medical Screening (IMS) at the Sheba Medical Center (SMC), Israel, at least twice during the years 2013–2019. The IMS offers an advanced medical screening program based on current international clinical practice guidelines [19, 20]. Most IMS patients have a high SES, generating a relatively homogeneous cohort, and reducing the impact of socio-economic confounders. Excluded were patients who had visited the IMS only once during those years, or possessed only one blood test. The first visit to the IMS for most study individuals occurred before 2013, with the earliest visits in 2001. The Institutional Review Board of the SMC, Israel approved the study (registered as #6327–19-SMC). A waiver of consent was obtained from the Institutional Review Board.

### Data source

Data, including all patient history, laboratory results and medications were obtained from the computerized database of the IMS. The patients’ diagnoses were retrieved from the Registry by applying diagnosis-specific algorithms, including the International Classification of Diseases, 9th version (ICD-9) code and text reading, laboratory test results and specific drug usage. Data on malignancies (type and date of diagnosis) of IMS patients was obtained from the National Cancer Registry, updated in November 1, 2019. Since cognitive tests were not part of the IMS protocol**,** the diagnosis of cognitive decline was obtained upon reviewing the medications prescribed for Alzheimer’s disease; results of the Mini Mental State Examination (MMSE); the Montreal Cognitive Assessment (MOCA) or the Mini COG Score, when available. Other patients were diagnosed according to the ICD-9 or free text of electronic medical charts. Notably, in Israel, MMSE is required prior to prescribing first time medications for Alzheimer’s disease patients. Patient mortality data were retrieved from the Israeli Ministry of Interior’s National Mortality Registrar.

### Evaluation of collected data

We conducted a thorough manual quality control assessment of all patients, identified potential discrepancies and enriched the data with comments written in free text or on scanned documents from years prior to 2016, when not all documents were computerized.

### Statistical analysis

Overall lipid levels for each individual were computed as the median of all the IMS results from 2013 to 2019. For analyzing survival, age was computed from birth until the end of 2019 or at death. Continuous variables were compared using Student t-tests when the normality assumption was appropriate, and by the Wilcoxon test for non-normal variables. The relationship between LLDs, lipid profile and the incidence of cognitive decline was examined using logistic regression, adjusting for age, hypertension, diabetes, smoking, stroke and ischemic heart disease (IHD). Cox proportional hazard regression models assessed the relationship between lipid measures and the use of LLDs to age at first onset of cancer, with observation from the first appointment at the IMS until cancer was diagnosed. Individuals who were not diagnosed with a malignancy were censored on November 1, 2019 or upon death, if occurring earlier. Participants whose first malignancy occurred prior to the first IMS visit were excluded. Analyses were performed for any type of malignancy and for 7 cancer categories: breast, lung, genitourinary, gastrointestinal, hematologic malignancy, melanoma and other cancer (e.g, head and neck, brain, thyroid, sarcoma, skin cancer requiring systemic therapy other than melanoma, or primary unknown). Participants diagnosed with a first malignancy in a different category, were treated as censored at the corresponding onset age. The model for breast cancer was limited to female participants (only one male); genitourinary cancers only to males (covering 147 of 152 malignancies). All Cox models were adjusted for smoking status (never, past, current), for sex (when both were included) and for polypectomies. Levels of TGs were transformed to a log scale for use in the Cox models. The model for any type of malignancy also included gender and its possible interaction with medication use. All *p*-values for medication and lipid profile were extracted and summarized using the false discovery rate (FDR) [21], with a 0.05 threshold for marking a discovery.

Cox models were also employed to analyze time to second malignancy. For these models, the time from first malignancy to second was taken as the event time. Excluded were those diagnosed with a second malignancy before the first IMS visit, but included those diagnosed with a single malignancy prior to the visit. The model was adjusted for smoking status, polypectomy and age at first malignancy.

## Results

### Clinical characteristics

Over 2000 patients (2175) were recruited patients who had attended the IMS during the years 2013–2019 and were ≥ 70 years of age at their last follow-up. According to the study protocol, 116 patients were excluded: 32 who had not performed a lipid profile test, 76 who had undergone only one lipid profile test and 9 who had visited the IMS only once. Overall, the cohort encompassed 1498 (72.7%) male and 561 (27.3%) female patients. The median age at the first visit was 59 for both men and women; 59.1 for those treated with LLDs and 58.9 for those not receiving any medication (*P* = 0.050, Wilcoxon test). Mean follow-up was 16.6 years for men and 15.6 years for women; 16.5 years for those on LLDs, 15.9 years for those not on LLDs (*P* < 0.001, Wilcoxon test). The mean and median age for patients who survived to the end of the study (i.e. November 2019), or age at death for those who died earlier, was 77.2 and 75.5, respectively for males and 76.1 and 74.4, respectively for females.

Detailed clinical characteristics of the patients are outlined in Table 1. Atherosclerotic risk factors e.g., hypertension, diabetes mellitus, dyslipidemia and smoking were found significantly more frequent in males. IHD and atrial fibrillation were more prevalent in males. Increased usage of anticoagulant or antiplatelet treatment was found amongst the males. Based on the National Institute of Health’s guidelines [22], our patients’ median body mass index (BMI) was in the lower range of the overweight category (Table 2). Fifty-one patients died during the follow-up; median age at death was 82.7 years. Crude death rates were 3.1% for males (46/1498) and 0.9% for females (5/561).

**Table 1:** Clinical characteristics of the cohort

Clinical characteristics	Men	Women		*P* value
Median age (mean)	75 (76.5)	75 (76.5)		
Hypertension (%)	1037 (67.2)	282 (50.3)		< 0.001
Diabetes mellitus (%)	422 (28.2)	70 (12.5)		< 0.001
Dyslipidemia (%)	1187 (79.2)	418 (74.5)		0.021
Smoking^*^ (past/active) (%)	811 (54.1)	230 (41.0)		< 0.001
Ischemic heart disease (%)	466 (31.1)	24 (4.3)		< 0.001
Stroke (%)	61 (4.1)	14 (2.5)		0.089
Cognitive decline (%)	88 (5.8)	35 (6.2)		0.756
Obstructive lung disease (%)	37 (2.5)	8 (1.4)		0.149
Chronic renal failure (%)	368 (24.6)	53 (9.5)		< 0.001
Atrial fibrillation (%)	165 (11.0)	33 (5.9)		< 0.001
Solid malignancy (%)	372 (24.9)	124 (22.1)		0.192
Hematological malignancy (%)	75 (5.0)	23 (4.1)		0.386
Any malignancy (%)	429 (28.6)	139 (24.8)		0.081
Colonic polypectomy (%)	597 (39.9)	164 (29.2)		< 0.001
Lipid lowering drug use > 3 years (%)	1077 (71.9)	344 (61.3)		< 0.001
Anti-platelet therapy (%)	772 (49.7)	140 (24.9)		< 0.001
Anti-coagulant therapy (%)	173 (11.6)	35 (6.2)		< 0.001
Death (%)	46 (3.1)	5 (0.9)		0.005

*Abbreviations*: *M* Males, *F* Females

^*^107 (7.1%) men and 30 (5.3%) women actively smoked, 704 (46.9%) men and 200 (35.7%) women were past smokers, 687 (45.9%) men, and 331 women never smoked (59.0%)

**Table 2:** Complete blood count results^a^ and BMI according to age and gender

Age group	70–75 (*N* = 973)		75–80 (*N* = 517)		80–85 (*N* = 345)		85+ (*N* = 224)	
Gender	Male	Female	Male	Female	Male	Female	Male	Female
Count	683	290	380	137	258	87	177	47
White blood cells^b^	6.5	5.9	6.5	6	6.6	6.2	6.5	6.5
Absolute neutrophil count^b^	3.9	3.4	4	3.7	4	3.6	4.1	3.9
Absolute lymphocyte count^b^	1.8	1.8	1.7	1.8	1.8	1.7	1.6	1.8
Hemoglobin (g/dL)	14.6	13.2	14.5	13.4	14.2	13.2	13.6	12.9
Mean corpuscular volume (fL)	89.9	97.1	90.4	89.9	91.4	90.7	91.4	89.9
Platelets^b^	203	204	199	206	197	207	202	216
Body mass index	27	27.6	26.5	25.5	26	25.1	25	25.2

^a^Data is presented as median for age groups

^b^Values in K/microL

### Laboratory findings

#### Lipid profile

**Fig. 1:**
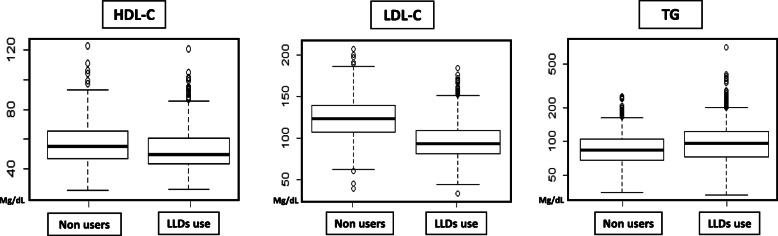
Lipid profile and lipid lowering drugs users versus non users. High density lipoprotein (HDL-C), low density lipoprotein cholesterol (LDL-C) and triglycerides (TGs) (median for individual), lipid lowering  drugs (LLDs)

The distribution of HDL-C, LDL-C and TGs in accordance with the use or non-use of LLDs is presented in Table 1(Suppl). Overall, 1421 (69%) patients were treated with LLDs: 1195 (84%) with statins alone; 162 patients (11.4%) with statins and ezetimibe and 24 (1.7%) with statins and bezafibrates. Twenty-eight (1.9%) patients were treated only with ezetimbe due to an intolerance to statins; one other patient was treated with a proprotein convertase subtilisin/kexin type 9 (PSCK9) inhibitor. Seven (0.49%) patients were treated only with bezafibrates due to hypertriglyceridemia. We further analyzed the difference in median values of HDL–C, LDL-C and TGs between LLD users and non-users (Fig. 1). Patients treated with LLDs exhibited a significantly lower HDL-C with a median of 48 mg/dL compared to 53 mg/dL in non-users, interquartile range (IQR) of 42–58 and 45–63, respectively, *p* < 0.001. These patients also exhibited a lower LDL-C with a median of 95 mg/dL compared to 125 mg/dL in non-users, IQR of 83–111 and 109–141, respectively, *p* < 0.001. Levels of TGs were higher in patients treated with LLDs with a median of 107 mg/dL compared to 94 mg/dL in non-users, but 10–15% lower for the first and third quartiles with an IQR of 82–138 and 76–118, respectively (*p* < 0.001).

#### Link between lipid profile and LLDs use later in life and cognitive decline

**Fig. 2:**
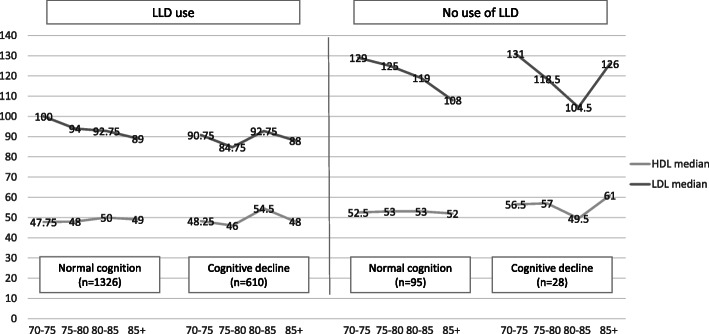
The effect of LDL-C, HDL-C and LLDs on cognitive impairment according to age. Data are presented as median for age groups

During the study years, we identified 123 (5.97%) cognitively impaired patients; 88 (71.5%) males and 35 (28.5%) females; only 31 of these patients provided MMSE, MOCA or mini COG Score to IMS; 21 additional patients were on Alzheimer’s medications (e.g., donepezil, exelon, cerebonin). An additional 2 patients were transferred to the Stuchynski Alzheimer Center at Tel Hashomer, Israel, in close proximity to the IMS. Twenty-four patients were referred by IMS senior physicians to a psychogeriatric clinic or neurology department due to memory decline and the remaining 45 were diagnosed with memory decline by the ICD-9 or free text in the electronic medical records. Overall, 6.3% of the subjects treated with LLDs (95/1421) versus 4.2% non-users (28/638), declined cognitively. The association between HDL-C, LDL-C, LLDs treatment and cognitive decline in relation to age is presented in Fig. 2. Cognitive decline was more prevalent amongst the older patients. We further adjusted for a number of vascular risk factors: hypertension, diabetes, smoking, stroke and IHD. The age effect was highly significant, with an adjusted OR of 1.09 per year (*p* < 0.001). Other significant covariates were stroke (aOR 2.13, *p* value 0.027) and diabetes mellitus (aOR 1.54, *p* value =0.042). The effects of HDL-C and LDL-C, when adjusting for the above covariates were not significant (*P* = 0.44 and 0.29, respectively). LLDs treatment was associated with an increased risk of cognitive decline, but not significant (aOR 1.35, 95% CI of 0.85–2.17, *P* = 0.21). Also when adjusting for HDL-C and LDL-C levels, the estimated OR was similar,1.29 (95% CI 0.78–2.12, *P* = 0.33).

#### The relationship between lipid profile, LLDs and cancer

The relationship of the lipid profile to carcinogenesis may differ due to the type of cancer. We, therefore, chose to analyze the overall impact of the lipid profile and LLDs and their impact on distinct groups of cancers. We identified 568 patients who presented with one type of malignancy. Of these, 406 patients (323 men; 83 women) were diagnosed after their first visit to the IMS and were included in subsequent analyses. The median age of the subjects upon entry to the study for those presenting without (with) a prior malignancy was 58.7 (64.5). Table 3 summarizes the types of malignancies and time of occurrence.

**Table 3:** List of malignancies diagnosed before or during active surveillance in the institute for medical screening

Malignancy category	1st malignancy diagnosed pre-IMS surveillance			1st malignancy diagnosed during IMS surveillance			Second and additional malignancies diagnosed during IMS surveillance		
	Men	Women	Total	Men	Women	Total	Men	Women	Total^†b^
Breast	0	22	22	1	39	40	1	4	5
Lung	2	1	3	15	3	18	1	1	2
Gastrointestinal^*^	16	5	21	40	4	44	8	0	8
Genitourinary^‡^	49	3	52	147	5	152	33	1	34
Hematologic^§^	12	3	15	56	14	70	5	7	12
Gynecologic^ǁ^	0	6	6	0	4	4	0	2	2
Melanoma	18	13	31	55	9	64	14	1	15
Other^¶^	8	3	11	9	5	14	5	5	10

^*^Gastrointestinal- colorectal, pancreas, biliary, stomach, esophagus and liver; ^**‡**^Genitourinary- prostate, kidney, urothelial, bladder, testicular; ^**§**^Hematologic- leukemia, Hodgkin’s and non-Hodgkin’s lymphoma, multiple myeloma and myelodysplastic syndrome; ^**ǁ**^Gynecologic- ovarian, uterine, cervical; ^**¶**^Other- head and neck, brain, thyroid, sarcoma, skin cancer requiring systemic therapy other than melanoma, or primary unknown; †15 cases of second malignancy were diagnosed prior to the IMS surveillance: 2 breast, 2 gastrointestinal, 3 genitourinary, 2 hematologic, 1 gynecologic, 4 melanoma and 1 other (advanced squamous cell carcinoma)

**Fig. 3:**
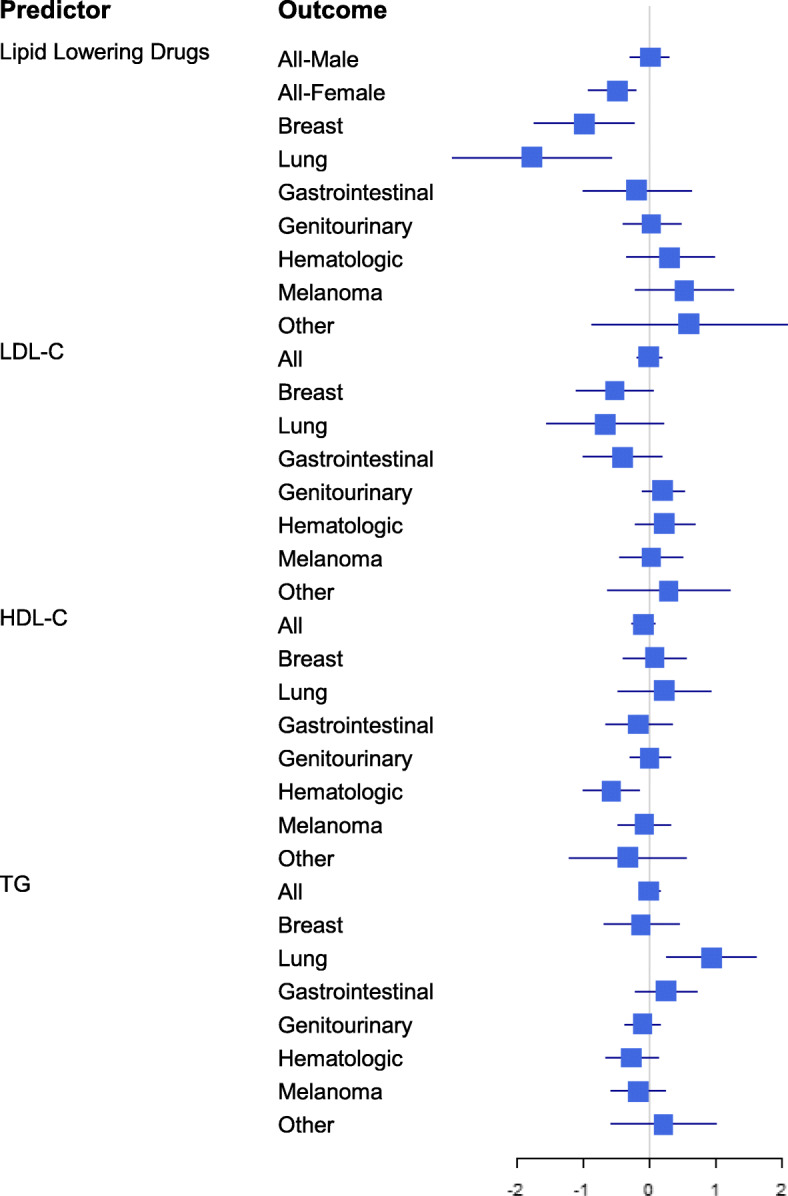
A forest plot with the log HRs for each cancer type according to LLDs, LDL-C, HDL-C, and TGs

Cox proportional hazard models were fitted for malignancies of all types and for 7 specific cancer groups. All models included effects for TGs, HDL-C, LDL-C and LLDs; the model for all malignancies included separate effects of LDL-C for males and females resulting in 33 estimated effects associated with LLDs or lipid profile measures. Figure 3 displays the log hazard ratios in a forest plot, with unadjusted 95% confidence intervals. The hazard ratio for LLDs compared those treated with the non-users. The hazard ratio for each lipid measurements relates to the difference defined by the IQR in our cohort. Application of the FDR to the corresponding *p*-values found that none of the effects were confirmed at an FDR threshold of 0.05. Three effects with *p*-values between 0.003 and 0.007 were close to that level (q = 0.071); a fourth effect with a *p*-value of 0.010, achieved q = 0.083. Three associations were best supported by the data: a strong protective association of LLDs with lung cancer (HR = 0.17); a protective association of higher HDL-C with hematologic cancers and an increased hazard of lung cancer with higher TGs. A protective association of LLDs with breast cancer (HR = 0.37) was also observed (*P* = 0.010).

The analysis of time to the second malignancy encompassed 550 patients; 66 presented with a second malignancy during the observation period. Another18 were diagnosed with a second malignancy prior to the IMS surveillance. The most common second malignancies were genitourinary (*n* = 34), and melanoma (*n* = 16). Second malignancies were diagnosed in 42/376 (11.2%) patients treated with LLDs compared to 24/174 (13.8%) non-users. The Cox model did not find a significant relationship between LLDs and time to second malignancy (*P* = 0.139) or for any of the lipid measures (LDL-C, *P* = 0.319, HDL-C, *P* = 0.241, TGs, *P* = 0.234). Amongst the adjustment factors, an increased hazard for second malignancy (Fig. 3) was found associated with older age at time of first diagnosis of malignancy (*P* = 0.011) and polypectomy (*P* = 0.024).

## Discussion

Life expectancy has increased over the past few decades [23] and despite developing age-associated diseases, many individuals live to > 100 years old [24]. Patients aged ≥70 with a high socio-economic background who had joined the IMS program were enrolled in this study. The high SES of patients may well have contributed to their longevity according to the Preston curve which showed a relationship between life expectancy at birth and income [25, 26], and the Tromso study which found that low income significantly affects survival [27]. The high rate of polypectomies in this cohort highlights their excellent overall level of health care. The incidence of hypertension, diabetes mellitus, IHD and cancer was surprisingly high in the male population, relative to the general Israeli population, aged > 65 according to the Israel National Health Interview Survey (INHIS-3) 2013–2015 [28]. Moreover, treatment with LLDs was higher in both men and women than reported in the INHIS-3 results. Most probably, early identification of lipid profile abnormalities hastened the initiation of LLDs treatment.

Despite the comorbidities of our cohort, the relatively normal hemoglobin values implied that our patients were neither frail nor anemic (Table 2). This is contrary to previous studies reporting that healthy, very elderly people usually present with lower hemoglobin levels [29]. Interestingly, the incidence of stroke and cognitive impairment was relatively low in our cohort. Since midlife hypertension, diabetes, smoking and obesity were found to be associated with an increased risk of cognitive decline [30], these confounders were included in the analysis of the IMS patients with cognitive decline. TGs were excluded due to the low number of patients with significant hypertriglyceridemia.

Although treatment with LLDs within the cohort was associated with a modest increase in the incidence of cognitive impairment after adjusting for age and other covariates, it was not statistically significant. Indeed, the real risk could not be accurately estimated due to the small sample size and the low incidence of cognitive decline. Interestingly, in a previous KOCOA project involving Japanese males and females aged > 80, higher LDL-C and lower TG/HDL-C ratios were found associated with higher scores in memory performance [31]. Research on Chinese individuals with a mean age of 85 years, found that cholesterol levels within the high normal range were associated with a better cognitive performance [32]. In a different community-based longitudinal study of Chinese living in a rural area where only ~ 0.4% had been treated with statins, lipid-cognition association was more pronounced in individuals aged 100 years. The authors postulated that this was the result of a faster cognitive decline rate in this age group [17].

When analyzing the association between HDL-C, LDL-C, TG, LLDs treatment and cancer, we found some effects on hazard, however, none were strong enough to pass our FDR filter. The strongest estimated effects were for LLDs (reduced hazard for lung and breast cancer) and for HDL-C (high levels associated with a reduced hazard for hematological malignancy). Yet, others have suggested that statin therapy does not influence cancer incidence [33] and that high levels of HDL-C protect people from developing non-Hodgkin’s lymphoma [8], whereas hypertriglyceridemia increases the risk of lung cancer [34]. This relationship was also found in our cohort.

### Mortality

Since the mortality in this cohort was low (51 deaths), and there was a need to control for a number of risk factors, we concluded that the data were insufficient at this time to permit an assessment of effects on mortality. A previous study focusing on elderly female patients found no association between LDL-C, HDL-C and longevity [35].

### What does the current work add to the existing knowledge

This study focuses on elderly patients of a high socioeconomic status who attended an advanced screening program for many years. The uniformity of the cohort reduced potential confounding related to socioeconomic diversity. The association of LLDs and cancer in the elderly was researched, at present, there is a dearth of data.

### Limitations and strengths

The strengths of this study were the uniformity of the cohort and the high quality of the clinical data collected. All members of the cohort had high SES and good health consciousness, limiting potential confounding due to socio-economic factors and health-seeking behaviors. However, this also limited the generalizability of our results. The clinical data were collected in a very uniform manner following the IMS protocol. Furthermore, all blood tests were analyzed at the same laboratory using the same assays. One of the study’s limitations was the study’s retrospective nature and as such, no precise date could be ascertained as to the beginning of the LLD treatment or for diagnosing conditions, such as diabetes mellitus, IHD, stroke, etc., or cognitive decline. Explicit data was lacking as to the reasons for initiating, or for declining to initiate LLD treatment, data on adherence to statin treatment [36] and depression status, as these were not part of the standard IMS protocol. The latter is relevant since a substantial depletion of cholesterol was reported to affect mood, especially, in the elderly and negatively affects performance on cognitive tests [37]. Another limitation was the missing MMSE or Mini Cog score for most patients which is partly related to the fact that the tests are not part of the IMS protocol. The cohort, although of reasonable size, did not include a sufficient number of events, in particular, death, cognitive decline and some types of cancer, to enable an accurate assessment of LLDs or lipid associations.

## Conclusions

Analysis of an elderly, high SES cohort with high quality data suggests that several relationships exists between the use of LLDs and health outcomes. Those treated with LLDs had a lower risk of lung and breast cancer, but a higher risk of cognitive decline. For all outcomes, the number of events was too small to reach statistical significance. These relationships warrant further study to assist the clinician in choosing the appropriate LLDs in the elderly.

## Supplementary Information

**Additional file 1.**

## Data Availability

Not applicable.

## Abbreviations

LDL-C: Low density lipoprotein cholesterol
HDL-C: High density lipoprotein cholesterol
LLDs: Lipid lowering drugs
GWAS: Genome-wide association studies
CVD: Cardiovascular disease
TGs: Triglycerides
IMS: Institute for medical screening
SMC: Sheba Medical Center
ICD-9: International classification of diseases, 9th version
IHD: Ischemic heart disease
BMI: Body mass index
CBC: Complete blood count
IQR: Interquartile range
